# Detection of viable *plasmodium* ookinetes in the midguts of *anopheles coluzzi* using PMA-qrtPCR

**DOI:** 10.1186/s13071-015-1087-8

**Published:** 2015-09-15

**Authors:** Tibebu Habtewold, Zoe Groom, Luc Duchateau, George K. Christophides

**Affiliations:** Department of Life Sciences, Imperial College London, London, UK; Department of Comparative Physiology and Biometrics, University of Ghent, Ghent, Belgium; Costello Medical Consulting, Cambridge, UK

**Keywords:** *Anopheles coluzzii*, Propidium monoazide, *Plasmodium*, Midgut invasion, Commensal microbiota

## Abstract

**Background:**

Mosquito infection with malaria parasites depends on complex interactions between the mosquito immune response, the parasite developmental program and the midgut microbiota. Simultaneous monitoring of the parasite and bacterial dynamics is important when studying these interactions. PCR based methods of genomic DNA (gDNA) have been widely used, but their inability to discriminate between live and dead cells compromises their application. The alternative method of quantification of mRNA mainly reports on cell activity rather than density.

**Method:**

Quantitative real-time (qrt) PCR in combination with Propidium Monoazide (PMA) treatment (PMA-qrtPCR) has been previously used for selectively enumerating viable microbial cells. PMA penetrates damaged cell membranes and intercalates in the DNA inhibiting its PCR amplification. Here, we tested the potential of PMA-qrtPCR to discriminate between and quantify live and dead *Plasmodium berghei* malarial parasites and commensal bacteria in the midgut of *Anopheles coluzzii* Coetzee & Wilkerson 2013 (formerly *An. gambiae* M-form).

**Results:**

By combining microscopic observations with reverse transcriptase PCR (RT-PCR) we reveal that, in addition to gDNA, mRNA from dead parasites also persists inside the mosquito midgut, therefore its quantification cannot accurately reflect live-only parasites at the time of monitoring. In contrast, pre-treating the samples with PMA selectively inhibited qrtPCR amplification of parasite gDNA, with about 15 cycles (Ct-value) difference between PMA-treated and control samples. The limit of detection corresponds to 10 *Plasmodium* ookinetes. Finally, we show that the PMA-qrtPCR method can be used to quantify bacteria that are present in the mosquito midgut.

**Conclusion:**

The PMA-qrtPCR is a suitable method for quantification of viable parasites and bacteria in the midgut of *Anopheles* mosquitoes. The method will be valuable when studying the molecular interactions between the mosquito, the malaria parasite and midgut microbiota.

## Background

The first hours after ingestion of an infectious blood meal by the mosquito vector are key for sucssesful infection with malaria parasites. This period of the *Plasmodium* lifecycle involves gametogenesis, fusion of the male and female gametes, zygote development and differentiation to motile ookinete, ookinete traversal of the peritrophic membrane and the midgut epithelium and transformation to oocyst. Importantly, this period is marked by a major drop of parasite numbers. The rate of parasite losses during the ookinete to oocyst transition in *An. gambiae* is shown to reach 50, 41 and 69 folds for *P. berghei* [1], *P. yoelii* [2, 3] and *P. falciparum* [4], respectively.

Inside the mosquito blood bolus, *Plasmodium* parasites are found in close proximity with the mosquito midgut microbiota. The *Anopheles* mosquito midguts harbour numerous genera of microbiota, including *Pseudomonas*, *Aeromonas*, *Asaia*, *Comamonas, Elizabethkingia*, *Enterobacter, Klebsiella*, *Pantoea*, *Serratia* and others [5]. These microbiota proliferate dramatically and reach a peak at approximately 24–30 h post blood meal, before falling back to pre-blood meal levels [6]. The microbiota in the mosquito midgut play a significant role in malaria transmission dynamics. It has been shown that *Plasmodium* infection intensity is increased when mosquitoes are treated with antibiotics and this phenotype is reversed when microbiota are reconstituted (e.g. [7, 8]). Interaction of the microbiota with *Plasmodium* development appears to be exclusive to certain strains of gram-negative bacteria [9]. Currently, the mechanisms underlying the apparent bacterial inhibition of *Plasmodium* development are not fully elucidated; they are thought to involve parasite killing both directly and through induction of mosquito immune responses [9–12]. Induction of immune responses either through the gut microbiota or through direct parasite recognition plays a major role in vector competence.

Microscopic examination of mosquito midgut epithelium remains the gold standard for determining *Plasmodium* infection intensity*.* Dissection and microscopic examination of mosquito midguts is typically performed 5–7 days after infection, in order to allow the blood meal bolus to clear and the oocyst to increase in size becoming detectable. However, by this time, the gut bacterial population and mosquito innate immune activity have subsided to a normal state. Thus, microscopy does not allow the simultaneous assessment of the parasite, bacterial and immune response dynamics. Quantitative real-time PCR (qrtPCR) of genomic DNA (gDNA) are currently used as a fast, sensitive, and specific molecular tool for the detection and quantification of pathogens, including *Plasmodium* parasites in the mosquito [13, 14]. However, false-positive results due to amplification of gDNA from dead cells are a major drawback. In environmental microbiology, an alternative method involves mRNA-based qrtPCR for selective detection of viable cells [15, 16]. This technique takes advantage of the rapid mRNA degradation compared to gDNA. However, mRNA does not accurately represent bacterial cell density but mostly refers to bacterial cell growth and proliferation.

A diagnostic technique that precludes detection of DNA from dead cells involves pre-treatment of samples with propidium monoazide (PMA) and subsequent PCR analysis of gDNA [17]. This method is referred as viability PCR (v-PCR). PMA has high affinity to double stranded DNA and, upon photolysis using bright white light, reacts strongly with hydrocarbons of the bound DNA inducing permanent modification and rendering it inaccessible to DNA polymerase [18]. The technique exploits the fact that PMA not only binds to free floating DNA but also the DNA of dead cells as it penetrates the damaged or permeable membranes of dead cells but not the intact membranes of viable cells. The PMA-qrtPCR technique has been increasingly used for the selective detection of viable bacteria [19, 20], fungi [21] and protozoa [22]. Here, we evaluated the potential of the PMA-qrtPCR technique to simultaneously quantify viable *Plasmodium* parasites and bacterial microbiota in the mosquito midgut. This method could be used to investigate the interactions between the mosquito vector, the gut microbiota and the *Plasmodium* parasite during the first critical hours of mosquito infection.

## Methods

### Ethics statement

The protocol for infecting mice with *P. berghei* and *P. yoelii* was approved and carried out at the Imperial College London under the UK Home Office License PPL70/7185 awarded in January 2010.

### Mosquito colonies and maintenance

The *Anopheles gambiae* strain N’gousso M-form (a laboratory-strain colonized in 2006 from field mosquitoes collected around Yaoundé, Cameroon), now formally named as *Anopheles coluzzii* Coetzee & Wilkerson 2013 [23], were used in these experiments. The mosquitoes were reared and maintained at 27 °C, 70 % relative humidity, subject to a 12 h light/dark cycle. Adult mosquitoes were fed on 10 % sucrose cotton pads.

### Plasmodium strains and maintenance

Green Florescent Protein (GFP)-*Plasmodium berghei* (ANKA 2.34 strain) [24] or GFP-*Plasmodium yoelii* (17X strain) [25] parasite lines were used throughout this study. The parasite lines were maintained by serial passage in 8–12 week-old female TO mice (Harlan, UK).

### Mosquito infection with plasmodium

Female mosquitoes, 3–5 days old, were infected with *Plasmodium* parasites using two methods: (1) direct feeding on gametocytemic mice as previously described [26] or (2) using the membrane feeder system with ookinetes. For the ookinete culture, mice were injected with *P. berghei* or *P. yoelii* infected 3 days after phenyl hydrazine treatment to encourage reticulocyte formation. 3 days post infection, 1 ml of blood was drawn from the mice and immediately transferred to a vented tissue culture flask containing 30 mL 80 % (v/v) complete ookinete culture medium (RPMI1640 (Sigma)). The culture was incubated at 21 °C in air for 24 h (48 h for *P. yoelii*). The number of mature ookinetes was determined with a haemocytometer. Blood meal serum, containing approximately 800 ookinetes/μl, was offered to mosquitoes for feeding.

### Injection of mosquitoes with ookinetes

Mosquitoes were directly injected with viable or heat inactivated ookinetes (heated at 42 °C for 15 min) into their hemolymph. Each mosquito received ~400 ookinetes in freshly prepared ookinete culture media. Mosquitoes challenged with *P. berghei* and *P. yoelii* parasites in all the above methods were maintained at 21 °C and 24 °C, respectively.

### Dissecting mosquito midgut and harvesting gut content

Midgut contents of infected mosquitoes were dissected on ice cold PBS + BSA (2 %). For harvesting the gut contents, a midgut was placed in 20 μl of PBS and a longitudinal incision was made on the gut to draw out its contents into the PBS. The resulting sheet of midgut tissue and the gut contents were used in subsequent microscopic examination or nucleic acid extraction.

### Preparation of samples for microscopic examination

Midgut sheets were immunostained with Pbs28 antibody conjugated with 13.1-Cy3 dye for 30 min on ice and then examined under florescent microscopy to detect or enumerate dead and viable ookinetes. Dead ookinetes were negative for GFP. Alternatively, midguts were subjected to dual staining using propidium monoazide (PMA) and Syber Green (Sb) I dyes. Cells with compromised membranes (considered as inactive) were positive for red florescence arising from PMA, whereas the viable cells remained green.

### Total RNA (tRNA) extraction

Total RNA was extracted from whole mosquito or midgut content. To extract tRNA from a whole mosquito, 10 mosquitoes were transferred into 2 ml Safe-Lock RNA/DNA extraction RNase free tube containing 90 μl lysis buffer (3 mg/ml lysozyme in TE) and ~25 ng acid washed glass beads. The mixture was then incubated in a thermostatic mixer for 10 min at 37 °C at 300 rpm, and for an additional 10 min after adding 10 μl proteinase K. Buffer RLT (350 μl) was added to the mixture, and then homogenised using Precellys® 24 tissue homogeniser. The homogenate was spiked with 106 copies of control RNA template transcribed from the plasmid pAW109 (GeneAmp® RNA kit) as an internal standard. Total RNA was also extracted from the midgut content of mosquito. In this case, the midgut was emptied into 20 μl PBS. The gut content together with PBS was transferred to an RNase free tube containing the lysis buffer (80 μl). After incubation (see above), the RLT buffer (350 μl) was added to the mixture and the mixture was spiked with control RNA template. For all types of samples in RLT buffer, QIAGEN RNeasy® Mini kit was used to extract tRNA following the kit protocol.

### Sample preparation for treatment with PMA

*In vitro* ookinete culture, bacterial culture from mosquito gut content, midgut epithelial tissue of mosquitoes, gut content and whole mosquitoes samples were prepared for PMA treatment as follows. The total volume of *in vitro* ookinete culture, containing a known number of mature ookinetes, was adjusted to 500 μl to prepare for PMA treatment. Bacterial culture was prepared for the PMA treatment as follows. The midgut bolus was removed from mosquitoes that had obtained a naive bloodmeal 24 h before the dissection and was directly inoculated into LB medium (5 ml) and incubated over night at 37 °C. A 500 μl aliquot of the bacterial culture was removed and heated at 100 °C for 10 min before PMA treatment. The control sample was left unheated. Ookinetes embedded in the midgut epithelia tissues were prepared for PMA treatment. First, midguts were dissected (*n* = 10) on ice cold PBS + PMA and were transferred into 500 μl ice cold PBS-BSA (2 %) after removing blood boluses. The bloodmeal bolus in PBS + BSA was transferred into a separate tube for PMA treatment. To prepare whole mosquitoes for PMA treatment, first a longitudinal incision was made in the abdomen to expose the interior including the midgut that was dissected onto ice cold PBS + BSA. Then an additional longitudinal incision was made into the midgut. All the mosquito tissue, including the blood meal bolus, was transferred to a tube and the volume was adjusted to 500 μl with PBS-BSA. Finally, the sample was placed on a rocker for 1 h in the cold and the circular sheath was carefully removed with fine forceps.

### PMA treatment and genomic DNA (gDNA) extraction

Procedures for PMA treatment described previously [19, 27] were used with minor modification. Briefly, PMA stock solution (20 mM in 20 % dimethyl sulfoxide; Biotium Inc., Hayward, CA) was added at a concentration of 200 mM. The sample-PMA mixture was incubated in the dark for 20 min. To cross link PMA to DNA molecules, the samples were placed horizontally on ice on a shaker and exposed for 5 min to a 650 W halogen lamp at a distance of 20 cm. Genomic DNA extraction was performed on the PMA treated samples using a QIAGEN DNeasy® Blood & Tissue kit. After PMA treatment, the sample was spun at 3000 rpm for 10 min at a cold temperature, and the supernatant was replaced by 180 μl of lysis buffer from the kit. This mixture was incubated for 30 min at room temperature and then for 30 min at 55 °C after adding 20 μl proteinase K. The mixture was then homogenised using Precellys® 24 tissue homogeniser. Before proceeding to the gDNA extraction, the homogenate was spiked with 106 copies of control cDNA synthesised from RNA template transcribed from the plasmid pAW109 (GeneAmp® RNA kit) as an internal standard. The QIAGEN DNeasy® Blood & Tissue kit protocol was followed to extract gRNA.

### Reverse transcription polymerase chain reaction (RT-PCR)

Expression of Secreted Ookinete Adhesive Protein (SOAP) gene by *Plasmodium* ookinetes was assessed using RNA isolated from the whole mosquito or from the blood meal bolus at specified time points. RT-PCR was performed using the QIAGEN One-step RT-PCR method which ensures a high specificity and sensitivity and minimises sample loss and contamination. Briefly, tRNA samples (25 μl) were added to an RT-PCR master mix containing 5x OneStep RT-PCR Enzyme Buffer 10 μl, dNTP mix (10 mM) 2 μl, 5x Q-Solution 10 μl, gene specific primers (10 μM) 0.5 μl each and One-step RT-PCR Enzyme mix 2 μl to make a final volume of 50 μl. The primers and their sequences used for amplification of the SOAP gene in both the parasite species were Forward, TCGAAGGAGCAAGGAAAAATTCC and Reverse, ATGAACAGCTACATTCTTCGGTC. The amplified product was a 438 bp fragment. Exogenous pAW109 RNA (106), co-extracted with the sample RNAs, served as an invariant control. The GeneAmp® RNA kit primers for pAW109 control RNA were used to amplify a product of 308 bp, primer DM151, GTCTCTGAATCAGAAATCCTTCTATC and Primer DM152, ATGTCAAATTTCACTGCTTCATCC were also used. The reaction mixture was first incubated at 50 °C for 30 min to reverse transcribe tRNA, followed by heating at 95 °C for 15 min to activate DNA Polymerase, deactivate Omniscript and Reverse Transcriptase, and denature the cDNA template. Initial heating was followed by 30 cycles of 94 °C for 30 s, 56 °C for 30 s and 72 °C for 30 s. The final elongation stage was carried out at 72 °C for 4 min. RT-PCR products were analysed by agarose 1 % gel electrophoresis.

### Real-time PCR (QRT-PCR)

A series of qrtPCR assays were performed on the gDNA that was extracted from *In vitro* ookinete culture, bacterial culture from the mosquito gut content, the midgut epithelial tissue of mosquitoes, the gut content and the whole mosquito to determine the relative abundance of *Plasmodium* genes or gut bacterial genes. Set primer pairs used to amplify the *SOAP, CTRP or GFP gene were SOAP* Forward CCAAAACAACAGGCCAAGAG and SOAP Reverse AACATCGGCCAATGGATTAC, CTRP Forward TGCAATGATGTTTGTGGTGATTT and CTRP Reverse TGGTGATACATTTCTGGTTCTTATTCTT, GFP Forward CCTGTCCTTTTACCAGACAACCA and GFP Reverse GGTCTCTCTTTTCGTTGGGATCT. Universal 16S bacterial primers sets (357f CTCCTACGGGAGGCAGCAG and 519r GTTTACCGCGGCAGCTG) were used to amplify a 162 bp fragment. The PCR reactions were performed in a total volume of 20 μl, containing 2 μl gDNA, 10 μl of 2x SYBR®premix Ex Taq (Takara), 0.2 μM of each forward and reverse primer specific to target genes or internal standard and 0.4 μl Rox reference dye (50x). Amplification and detection of the florescence signal was carried out using an Applied Biosystems 7500 Fast Real-Time PCR system. The PCR cycling program consisted of an initial denaturation at 95 °C for 20 s, followed by 40 cycles of 95 °C for 3 s and 60 °C for 30 s. Each target was quantified in duplicate and the values were normalized by the data obtained with *An. gambiae* rRNA gene (AgS7) as internal standards. The primer sequences for the internal standard AgS7 gene were: forward, GTGCGCGAGTTGGAGAAGA and reverse, ATCGGTTTGGGCAGAATGC.

A standard curve was generated after qrtPCR analysis on a serial dilution of ookinetes from *in vitro* culture to calculate the limit of detection (LoD) and limit of quantification (LoQ) for PMA-qrtPCR techniques as described in a previous study [28].

## Results

### Microscopic and RT-PCR detection of ookinetes in the blood bolus

**Fig. 1:**
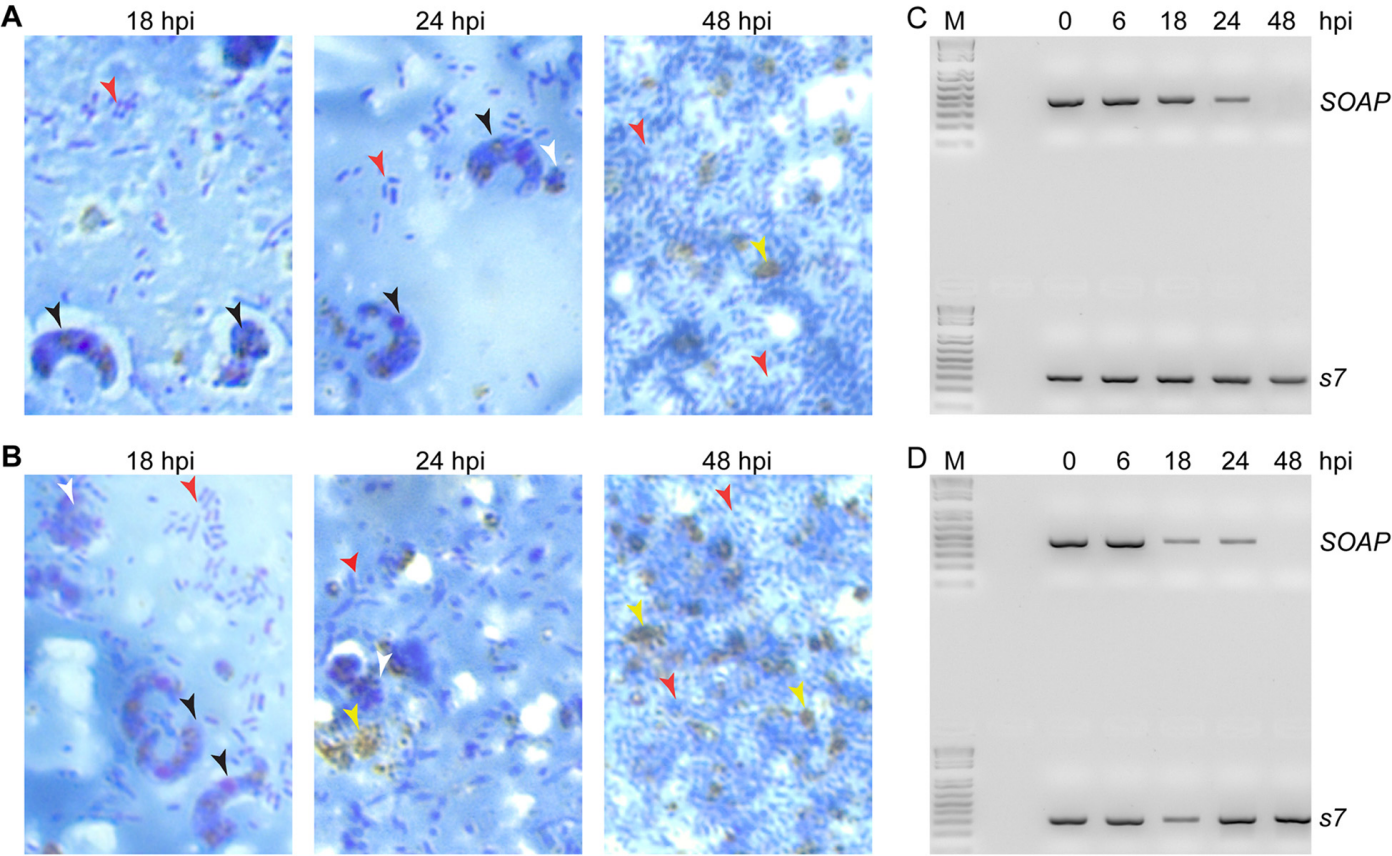
Detection of *Plasmodium* ookinetes in the mosquito midgut. **a** and **b** Representatitve images of smears of midgut contents stained with Giemsa from *An. coluzzii* mosquitoes infected with *P. berghei* (**a**) and *P. yoelii* (**b**). Midguts were dissected at 18, 24 and 48 h post infection (hpi). Arrowheads show zygotes/ookinetes (black), bacteria (red), remains of dead parasites (white) and blood meal contents (yellow). **c** and **d** Agarose gel images showing SOAP gene transcripts amplified from gut contents of mosquitoes infected with *P. berghie* (**c**) or *P. yoelii* (**d**). Midguts were dissected at 0, 6, 18, 24 and 48 hpi. The *An. gambiae S7* gene served as a heterologous internal standard. M corresponds to molecular marker

Direct microscopic examination of smears of midgut contents in *P. berghei* (Fig. 1a) and *P. yoelii* (Fig. 1b) infected *A. gambiae* mosquitoes confirmed that both zygotes and mature ookinetes were present in the gut lumen at 18 h post infection (hpi), while mature ookinetes were only observed at 24 hpi. All the ookinetes were physically cleared from the gut lumen by 48 hpi. However, RT-PCR performed on total RNA extracted from the mosquito midgut content showed that transcripts of the ookinete-specific gene *SOAP* were detectable until 48 hpi (Fig. 1c). The physical as well as molecular detection of *P. yoelii* ookinetes in the mosquito gut content were considerably more transient compared to *P. berghei*. This is expected as the *P. yoelii* infected mosquitoes were kept at a higher temperature (24 °C) compared to *P. berghei* (21 °C). Mature *P. yoelii* ookinetes were observed in midgut contents harvested at 18 hpi, but only ookinete remains were detected at 24 hpi. As with *P. berghei*, *P. yoelii SOAP* transcripts were still detectable at 24 hpi and only cleared by 48 hpi (Fig. 1d). These data indicate that ookinete-specific transcripts persist in the blood bolus even in the absence of viable ookinetes.

### Persistence of ookinete mRNA in the mosquito midgut and hemolymph

**Fig. 2:**
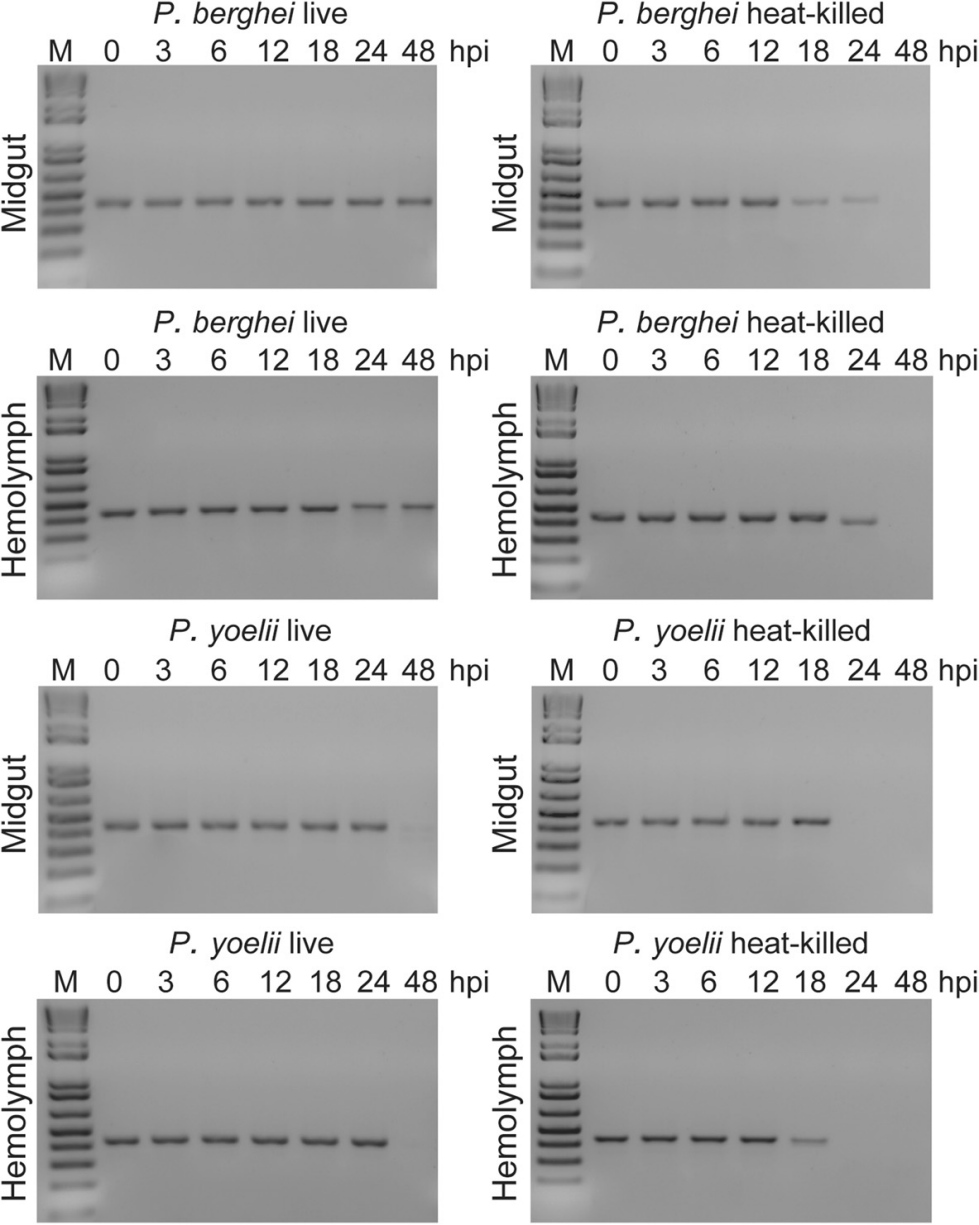
*SOAP* mRNA persistence in the *An. coluzzii* midgut and hemolymph. Agarose gel electrophoresis images of *Plasmodium SOAP* gene transcrips in *An. coluzzii* mosquitoes. Heat-killed or live *P. berghei* or *P. yoelii* ookinetes were delivered to mosquitoes either by addition to the blood meal or injection into the haemolymph. RT-PCR assays were carried our on whole mosquito RNAs prepared at 0, 3, 6, 12, 18, 24 and 48 h post ookinete ingestion or injection (hpi)

To determine the persistence of ookinete mRNA in mosquito tissues, heat-killed (42 °C for 30 min) or live *in vitro* cultured ookinetes were either added to mouse blood and offered to female *An. gambiae* mosquitoes as a blood meal via membrane feeding or injected directly into the mosquito hemolymph. Approximately 800 ookinetes were delivered in each mosquito with either of the two methods. RT-PCR analysis of *SOAP* transcripts was performed using total RNA extracted from whole mosquitoes at 0, 3, 6, 12, 18, 24 and 48 hpi. The results showed prolonged persistence of *SOAP* transcripts from both heat-killed and live *P. berghei* ookinetes compared to *P. yoelii* ookinetes (Fig. 2). This difference is attributable to the difference in temperature between these two infection models and consequently the slower metabolic rate of mosquitoes at 21 °C (*P. berghei*) compared to 24 °C (*P. yoelii*). For heat-killed ookinetes, *SOAP* transcripts persisted in mosquito tissues up to 24 hpi and 18 hpi for *P. berghei* and *P. yoelii* ookinetes, respectively, whereas transcripts from live ookinetes were detectable until 48 hpi. The slow mRNA decay rate suggests that transcript abundances measured by PCR methods are not fully reflective of viable parasites.

### PMA-induced inhibition of DNA amplification from dead parasites and bacteria

**Fig. 3:**
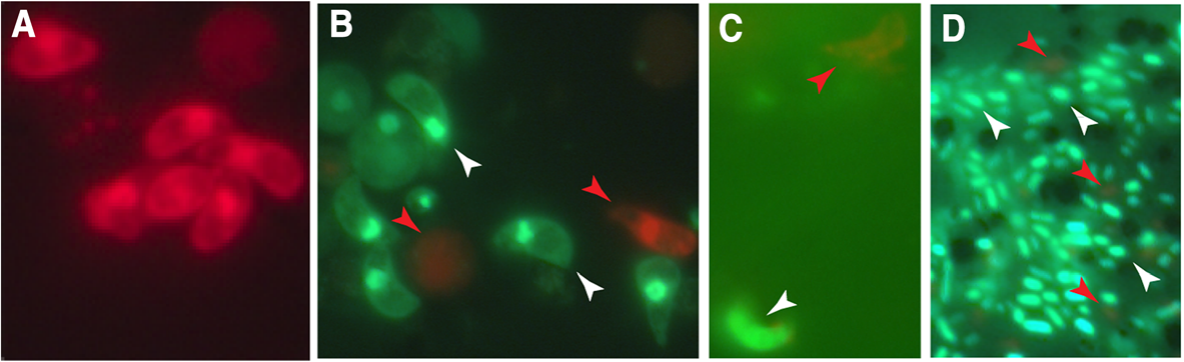
Differential staining of live and dead ookinetes and bacteria in the mosquito midgut. **a** Heat killed ookinetes an overnight ookinete culture stained with a mixture of PMA and SYBR solution. **b** Live and dead parasites from an overnight ookinete culture stained with a mixture of PMA and SYBR solution. **c** Live and dead *P. berghei* ookinetes in the mosquito midgut epithelium at 48 hpi. **d** Live and dead bacteria present in midgut contents. Live cells are stained with SYBR green (white arrowheads), while dead cells are stained with PMA (red arrowheads)

Propidium monoazide (PMA) has been increasingly used for the treatment of microbiological samples to exclude PCR signals from non-viable cells, but this has not been tested to date in investigations involving *Plasmodium*. We confirmed that PMA can stain dead *P. berghei* ookinetes prepared from an ookinete culture and heat-killed at 42 °C for 30 min before incubating them with PMA (Fig. 3a). Next, we stained ookinetes sampled directly from an overnight culture that was thought to include both live and dead cells. The ookinetes were incubated with a mixture of PMA and SYBR green that also stains nucleic acids but, unlike PMA, actively enters live cells. Fluorescent microscopic observations confirmed that some ookinetes are stained only with PMA, while most of the ookinetes are stained only with SYBR green (Fig. 3b). We applied the same protocol to *An. gambiae* midguts tissues infected 24 h earlier with *P. berghei*. The results showed that SYBR and PMA could be used to discriminate between live and dead parasites, respectively (Fig. 3c). Bacteria present in the blood meal bolus were also differentially stained with SYBR and PMA (Fig. 3d).

**Fig. 4:**
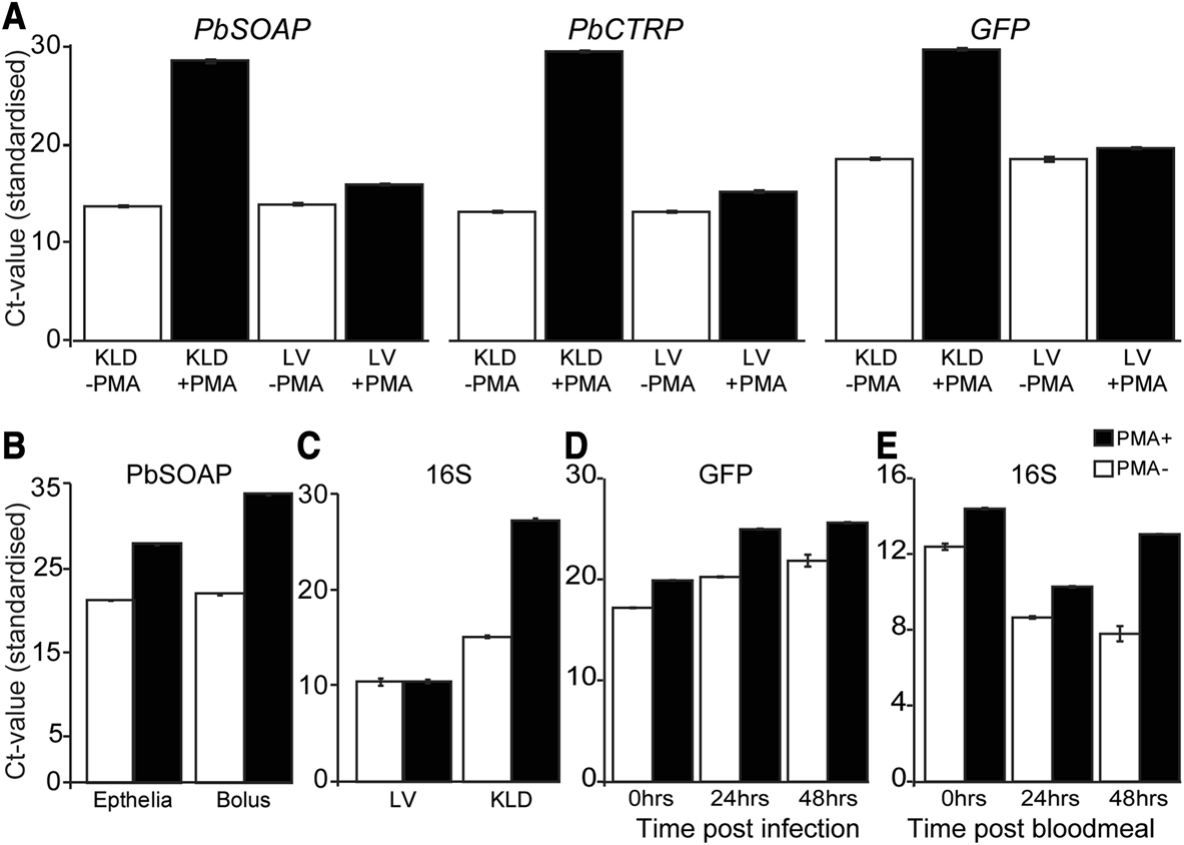
PMA-qrtPCR investigation of the inhibition of DNA amplification in killed ookinetes or bacteria during PCR. **a** Average cycle threshold (ct-value) for *Pb*SOAP *Pb*CTRP and *Pb*GFP_*CON*_ genes in heat inactivated (KLD) or live (LV) ookinetes from overnight gametocyte culture that were pre-treated with PMA. Inactivated and live ookinetes without PMA were used as a control. Samples, before DNA extraction, were spiked with an equal volume of *Anopheles coluzzii* homogenate with the aim to use the mosquito S7 gene as a heterologous internal standard. **b** Average ct-values for the *Pb*GFP gene in the midgut contents or gut epithelium tissue from mossquitoes 24 h pi with *P. berghei* parasite. **c** Average ct-values for bacterial 16S rRNA gene from heat inactivated samples and live bacteria cultured from mosquito midgut contents (24 h after bloodmeal). Samples without PMA treatment served as a control. **d** Ct-values for *Pb*GFP genes from gDNA samples that were extracted from mosquitoes that obtaned *P. berghei* ookinetes in their bloodmeal. **e** Ct-values for 16S rRNA genes in gDNA extracted from mosquitoes that obstained naïve bloodmeal. *An. gambiae S7* gene served as a heterologous internal standard for all the PMA-qrtPCR reactions that were performed using samples that were isolated from mosquito tissues. Error bars represent standard deviation from at least two independent assays. Each qrPCR experiment repeated three times

We assessed whether incorporation of PMA in nucleic acids could inhibit the PCR amplification rates of mRNA transcripts that derived from dead *P. berghei* parasites. Genomic DNA (gDNA) was prepared from heat-killed parasites from an overnight *in vitro* ookinete culture treated with PMA or control solution, and the abundances of *SOAP*, *CTRP* and *GFP* (the parasite line used carried a *GFP* expression cassette) amplicons were determined by quantitative real-time PCR (qrtPCR). The parasite gDNA samples were spiked with equal amounts of *An. gambiae* gDNA and amplification of the S7 gene served as a heterologous internal standard. The results showed that PMA treatment significantly inhibited the qrtPCR detection of *SOAP*, *CTRP* or *GFP* genes in dead ookinetes (Fig. 4a). We observed a similar inhibitory effect of PMA on qrtPCR assays for bacterial 16S rRNA gene in the gDNA from total culturable bacteria in the mosquito midgut content (Fig. 4b).

Next, we tested the PMA inhibitory effect on parasite detection in mosquitoes by comparing the level of reduction of qrtPCR signal (increase in Ct-value) in the midgut contents and midgut epithelial tissues of mosquitoes that had been infected 24 h earlier with *P. berghei* parasites. The qrtPCR analysis of the *PbSOAP* gene showed an increased Ct-value in gut content or gut epithelial tissue samples that were treated with PMA when compared to controls (Fig. 4c). The Ct-value was higher for PMA treated samples at 24 h pi compared to 0 h pi, but there was no further increase from 24 h to 48 h, suggesting the PMA treatment inhibited DNA amplification from dead ookinetes and also from those ookinetes that were eventually killed.

### Detection and quantification of ookinetes using PMA qrt-PCR

**Fig. 5:**
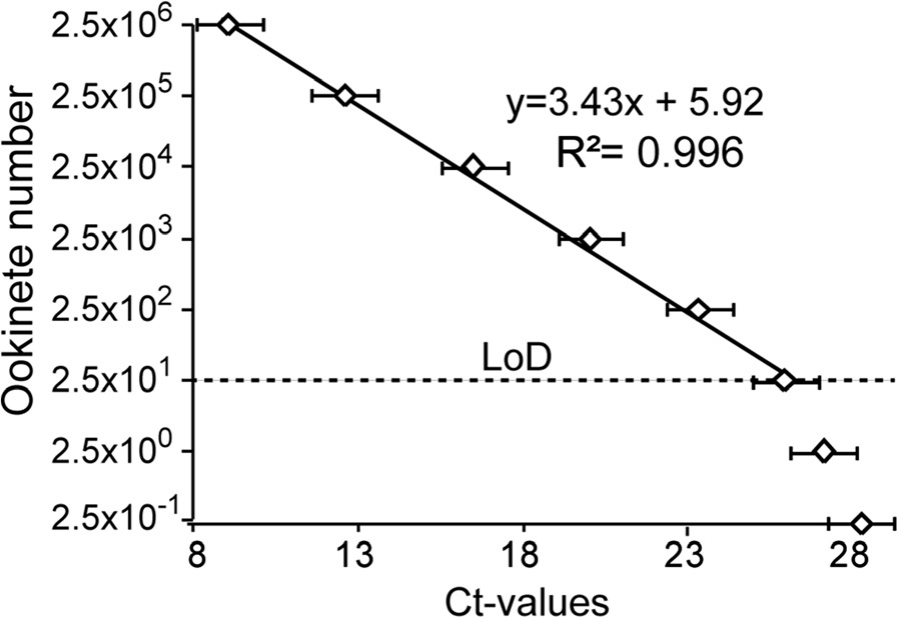
Ookinete detection efficiency using PMA-qrtPCR. Standard curve for quantifying *P. berghei* ookinetes using 10-fold serially diluted parasite cultures. Ookinete numbers are plotted against cycle threshold (Ct) values. Duplicate samples from two independent experiments were quantified. The dotted line indicates the limit of detection (LoD)

The efficiency of PMA-qrtPCR for detecting and quantifying *Plasmodium* ookinetes was tested using 10-fold serial dilutions of *P. berghei* ookinetes cultured *in vitro*, from 2.5×10^6^ to 2.5×10^−1^. Samples were spiked with equal volumes of mosquito tissue homogenates before proceeding to gDNA extraction. In two independent PMA-qrtPCR experiments, the resulting Ct-values were standardized to the *An. gambiae* S7 gene in the corresponding experiment. Averaged Ct-values corresponding to each dilution were plotted against the ookinete numbers in the respective dilution. Standard curves were linear for the serial dilution range (Fig. 5). The limit of quantification (LoD) for the PMA-qrtPCR was ~25 ookinetes.

## Discussion and Conclusions

In this study, we performed a series of experiments aimed to establish an efficient PCR based method to detect and quantify the success of *Plasmodium* parasite infection of the mosquito midgut. The majority of *Plasmodium* ookinetes are killed during the first 32 h of their life in the mosquito midgut. Dead parasites limit the use of standard PCR based methods to quantify live ookinete numbers during that time due to amplification of nucleic acids from both dead and viable parasites. An alternative method that addresses this limitation is quantification of mRNA that is often used as a viability marker on the basis of its rapid degradation and therefore considered to be a good indicator of live cells [29–32]. However, our data show that *Plasmodium* transcripts exclusively expressed in ookinetes and required for midgut invasion [33, 34] persist in mosquito midguts at least 24 h beyond the termination of their expression or after ookinete death. Therefore the detection and quantification of ookinetes transcripts using qrtPCR cannot exclude dead parasites, and mRNA originating from non-dead ookinetes in the midgut killed by multiple factors could lead to an overestimation of the number of ookinetes. Numerous studies have demonstrated that RNA can persist for up to a week in dead cells [35, 36]. Due to indecisiveness of discrimination of viable cells by this technique, the use of mRNA based qrtPCR is more adapted for gene expression studies than estimating of the bacterial abundance [37].

To circumvent the indecisiveness of mRNA-based techniques, a combination of pretreatment of samples with PMA and nucleic acid detection methods have been developed as an alternative strategy [38]. This tecnique is based on DNA detection of cells with intact cell/wall membranes, the viability qPCR (v-qPCR). The PMA selectively enters dead cells and interacts with DNA preventing its amplification during PCR. Until now, the PMA-qrtPCR technique has not been tested to date in investigations involving *Plasmodium* viability in the mosquito vector. We demonstrate that PMA can selectively enter dead ookinetes both from *in vitro* cultures and *in vivo*, while dual staining of the mosquito midgut contents with SYBR green I (SGI) and PMA confirmed that PMA enters exclusively into dead bacterial cells. Subsequent qrtPCR of PMA treated samples effectively differentiates viable from non-viable ookinetes, and the same effect is observed on bacteria isolated from mosquito midguts.

There are several examples of successful application of the PMA-qrtPCR technique including quantification of viable oocysts of *Cryptosporidium parvum* [22] and trophozoites and cysts of *Acanthamoeba castellani* [39]. The technique has been also successfully used to quantify viable microorganisms in environmental [40–42], food [43–45] and pathological samples [46, 47]. It has been shown that the limit of detection of the PMA-qrtPCR method was at least equivalent to that obtained by using CFU values for the gram-negative bacteria *Legionella pneumophila* [48]. Here we show that the technique can detect as few as 10 and accurately quantify as few as 25 ookinetes.

Therefore, the PMA-qrtPCR technique can allow accurate and simultaneous monitoring of viable *Plasmodium* ookinetes and midgut microbiota cells during the time of mosquito midgut invasion. The ookinetes and microbiota data obtained by this method can be also used to assess the dynamics and magnitude of mosquito immune responses at a time of infection.

## Abbreviations

PMA: Propidium Monoazide
PCR: Polymerase chain reaction
RT-PCR: Reverse transcriptase PCR
qrtPCR: Quantitative real-time PCR
DNA: Deoxyribonucleic acid
RNA: Ribonucleic acid
v-PCR: Viability PCR
SOAP: Secreted ookinete adhesive protein
PbSOAP: *Plasmodium berghei* SOAP
CTRP: Circumsporozoite and TRAP related protein
GFP: Green fluorescent protein
Ct: Threshold cycle
LoD: Limit of detection
